# Entomophytophagy ('Sequential Predatory, then Phytophagous Behaviour') in an Indian Braconid ‘Parasitoid’ Wasp (Hymenoptera): Specialized Larval Morphology, Biology and Description of a New Species

**DOI:** 10.1371/journal.pone.0156997

**Published:** 2016-06-29

**Authors:** A. P. Ranjith, Donald L. J. Quicke, U. K. A. Saleem, Buntika A. Butcher, Alejandro Zaldívar-Riverón, M. Nasser

**Affiliations:** 1 Insect Ecology and Ethology Laboratory, Department of Zoology, University of Calicut, Kerala, Pin: 673635, India; 2 Department of Zoology, Malabar Christian College, Calicut, Kerala, Pin: 673001, India; 3 Department of Biology, Faculty of Science, Chulalongkorn University, Phayathai Road, Pathumwan, BKK 10330, Thailand; 4 Colección Nacional de Insectos, Instituto de Biología, Universidad Nacional Autónoma de México, 3er. circuito exterior s/n Cd. Universitaria, Copilco, Coyoacán, A. P. 70–233, C.P. 04510, D.F., México; Indian Institute of Science, INDIA

## Abstract

The vast majority of braconid wasps are parasitoids of other insects. Although a few cases of pure phytophagy (primary gall production and seed predation) are known, no previous entomophytophagous species (i.e. ones that display entomophagy and phytophagy sequentially), has been discovered among braconids. We describe the detailed biology and specialized larval morphology for the first confirmed entomophytophagous braconid species. Leaf galls on *Garuga pinnata* Roxb. (Burseraceae) in India, induced by the psyllid, *Phacopteron lentiginosum* Buckton (Hemiptera: Psylloidea, Phacopteronidae) were sampled throughout a period of several months and found to suffer a high level of attack by a new species *Bracon garugaphagae* Ranjith & Quicke which is here described and illustrated. The wasps oviposit singly into the galls without paralysing the psyllids. The larvae first attack psyllid nymphs which they seek out within the gall, kill them with a single bite and consume them. Unique dorsal abdominal tubercles, with eversible tips present on the abdominal segments of the larvae that are used to help maintain larval position while feeding, are illustrated. After consuming all available prey, the larvae continue feeding on gall tissue until mature enough to spin cocoons and pupate. The new species illustrates, for the first time, a possible intermediate stage in the evolution of pure phytophagy within the Braconidae. Interestingly, the two unrelated seed predator *Bracon* species are also associated with Burseraceae, perhaps indicating that this plant family is particularly suited as a food for braconine wasps.

## Introduction

Braconid wasps represent one of the most diversified groups of insects comprising 46 subfamilies with nearly 1000 genera and 15,000 described species, the vast majority of which are parasitoids of other insects [1] and have been extensively used as experimental models of host–parasite associations [2–7]. Phytophagy in the Braconidae was first discovered in the subfamily Doryctinae with various species in a small number of genera being found to be primary gall formers [8–12]. Gall induction in the Doryctinae appears to be phylogenetically associated with parasitism of gall formers, but nothing is known of the transitional stages. Since then, primary gall formation has also been demonstrated in the genus *Mesostoa* (Mesostoinae) [13] whilst purely phytophagous seed predation is known in two Neotropical species of *Bracon* (Braconinae) [14, 15].

Members of the Braconinae, which is one of the largest of the subfamilies, are mostly ectoparasitoids that develop on concealed hosts that are usually paralysed as a result of venom injected by the female at the time of oviposition [16]. It is dominated by the genus *Bracon* which has more than 850 described species though molecular data strongly indicate that the genus is paraphyletic and possibly even polyphyletic [17]. Not surprisingly the host range of ‘*Bracon*’, is also by far the largest in the subfamily, and includes Coleoptera, Lepidoptera, Diptera [16], Hemiptera [18] as well as phytophagous Hymenoptera [19–21]. Almost all of these hosts share a moderate degree of concealment, usually in living plant tissues, and typically include inhabitants of tree bark, stems of annual and biennial plants, galls, seed heads or vessels, as well as leaf rollers, leaf miners and case-bearers [16].

Here we describe the biology of a new Indian braconid wasp species, *Bracon garugaphagae* sp. nov., whose larvae are initially predators of leaf gall-inducing psyllids (Hemiptera: Psylloidea) on *Garuga pinnata* Roxb. (Burseraceae). After the primary gall makers are consumed, they complete the greater part of their larval development consuming gall tissue. This is the first known instance of entomophytophagy [22] in the family. The wasp’s larval morphology includes several unique adaptations to this way of life.

## Materials and Methods

### Ethics statement

Necessary permits to conduct sampling of leaf galls were obtained from the Government of Kerala, India.

### Study site

This study was carried out in two sites in Malappuram district, Kerala, south India; viz. Kottakkal (10°99’N, 76°00’E) and Vettichira (10°93’N, 76°02’E). The sites have a tropical climate and during the study period the average annual temperature was 26°C and annual rainfall was 2842 mm [23].

### Sampling and data collection

Weekly field surveys were carried out from August 2014 to January 2015. Leaf galls induced by the psyllid, *Phacopteron lentiginosum* Buckton (Phacopteronidae), on shrubs of *Garuga pinnata* Roxb. were collected at different developmental stages and dissected under a stereomicroscope (Olympus).The number of gall-inducing nymphs and the presence or absence of braconid eggs, and early and mature larvae were recorded. Digital photographs of dissected galls and braconid wasp developmental stages were taken in situ with a Canon IXUS 255 HS digital camera.

### Species description

Alcohol-preserved specimens were processed with hexamethyldisilazane and later card-mounted. Images of the holotype and the egg, larva and pupa of the braconid wasp were taken with a Leica DFC 295 camera attached to a Leica S8 APO Stereozoomtrinocular microscope (Leica, Heerburg, Switzerland). Image stacks were combined into a single image and measurements of the holotype were done using Leica Application Suite V4.2. Images were edited using Photoshop CS8 (Version 6.1) (Adobe Inc.).

Morphological terminology employed in the description follows van Achterberg [24, 25] except wing venation nomenclature which follows Quicke [7]. Terms for sculpturing follow Eady [26] and Harris [27].

### Scanning electron microscopy and light microscopy of larvae

Larvae of different stages were dried with hexamethyldisilazane, mounted on entomological minuten pins and glued with epoxy resin on to standard electron microscopy stubs, sputter-coated with gold and studied under a JEOL JSM-5410LVmicroscope.

Larval head capsules were prepared from the gold-coated specimens as well as from uncoated material by macerating in 0.2M aqueous KOH to remove soft tissues, washing in dilute acetic acid, followed by dehydration through to xylene and mounting in Permount®.

### Molecular protocol

A specimen was sequenced for the barcoding 5’ fragment of the mitochondrial cytochrome oxidase gene and the nuclear 28S rDNA D2–D3 region following the methods of Zaldivar-Riverón et al. [28, 29]. Sequences were deposited in GenBank with accession numbers CNIN2004 KT343804 and CNIN2004 KT343805.

### Data analysis

All statistical analyses were carried out using the software package R [30].

### Nomenclatural Acts

The electronic edition of this article conforms to the requirements of the amended International Code of Zoological Nomenclature (ICZN), and hence the new names contained herein are available under that Code from the electronic edition of this article. This published work and the nomenclatural acts it contains have been registered in ZooBank, the online registration system for the ICZN. The ZooBank Life Science Identifiers (LSIDs) can be resolved and the associated information viewed through any standard web browser by appending the LSID to the prefix “http://zoobank.org/”. The LSID for this publication is: urn:lsid:zoobank.org:pub:FF0DA8B1-FB69-4203-8D37-0A6BDA5A2B64. The electronic edition of this work was published in a journal with an ISSN, and has been archived and is available from the following digital repositories: PubMed Central, LOCKSS

## Results

### Biology

**Fig 1:**
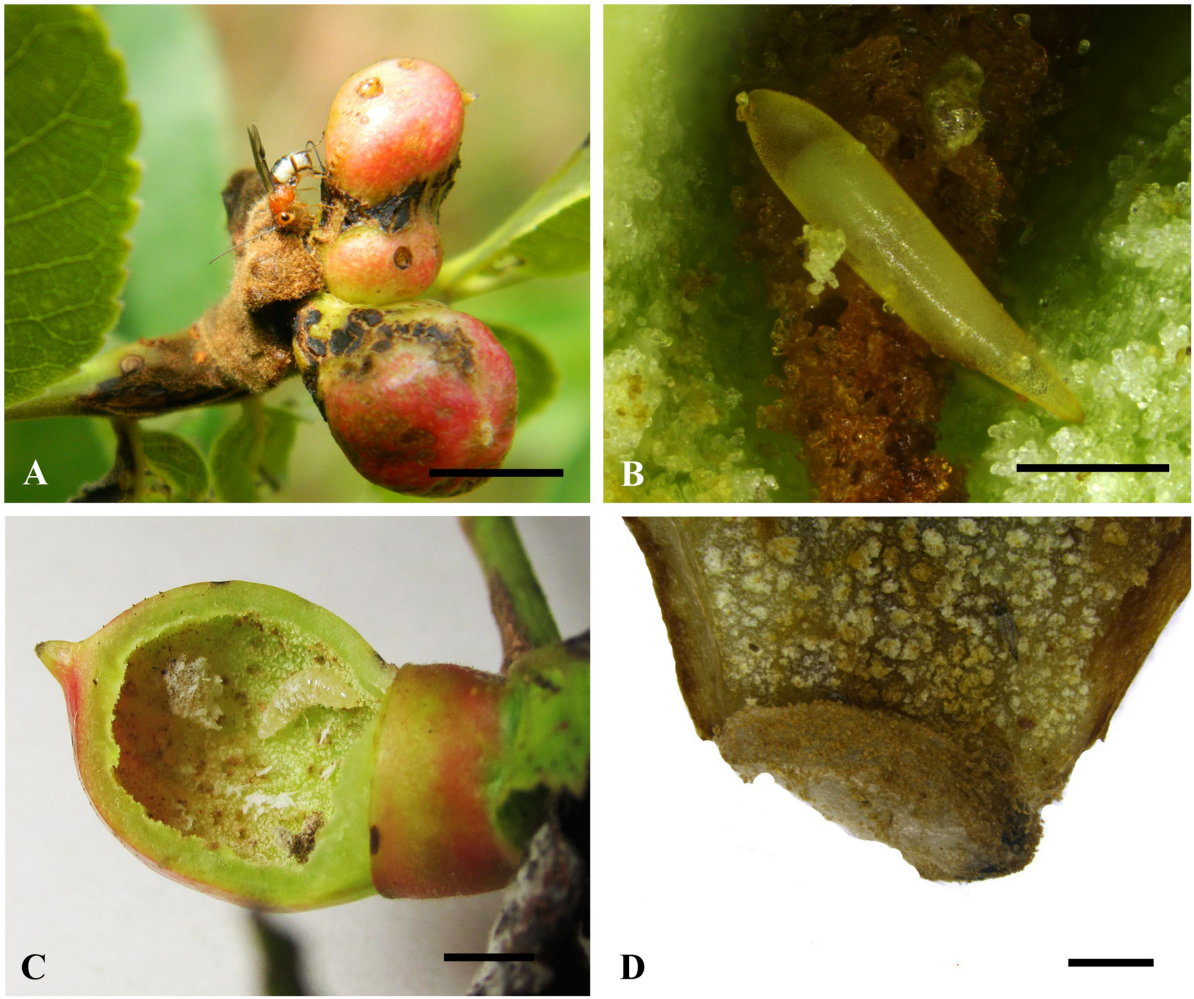
**Biology of *Bracon garugaphagae* Ranjith & Quicke sp. nov.** 1A, Adult female wasp ovipositing into gall induced by the psyllid, *Phacopteron lentiginosum* on leaf of *Garuga pinnata*. 1B, Braconid egg in situ.1C, Larva in situ in cut open gall. 1D, Cocoon attached to inner wall of gall. Scale bars: (1A) 8 mm, (1B) 300 μm, (1C) 5 mm, (1D) 1 mm.

Adult females of *B*. *garugaphagae* oviposit in moderately well-developed leaf galls (8–12 mm in diameter) on *G*. *pinnata* (Fig 1A). Eggs are 0.8–0.9 mm in length (n = 9), pale yellow green, spindle-shaped, having a short tail end, and are deposited on the inner wall of the floor of the gall chamber (Fig 1B) which is often associated with a white waxy substance. Larvae of *B*. *garugaphagae* were observed in galls ranging from 8–22 mm in diameter (n = 779). We did not manage to determine the precise number of instars. Maximum larval length was 5.3 mm (Fig 1C) (n = 779), at which stage larvae started to spin a cocoon (Fig 1D). The spindle-shaped, pale brown cocoons (3.5–3.7 mm long; n = 13) are constructed at the base of the gall chamber with the head end facing towards the gall floor. Adult wasps emerge from the gall by chewing an exit hole with their mandibles.

### Larval morphology

**Fig 2:**
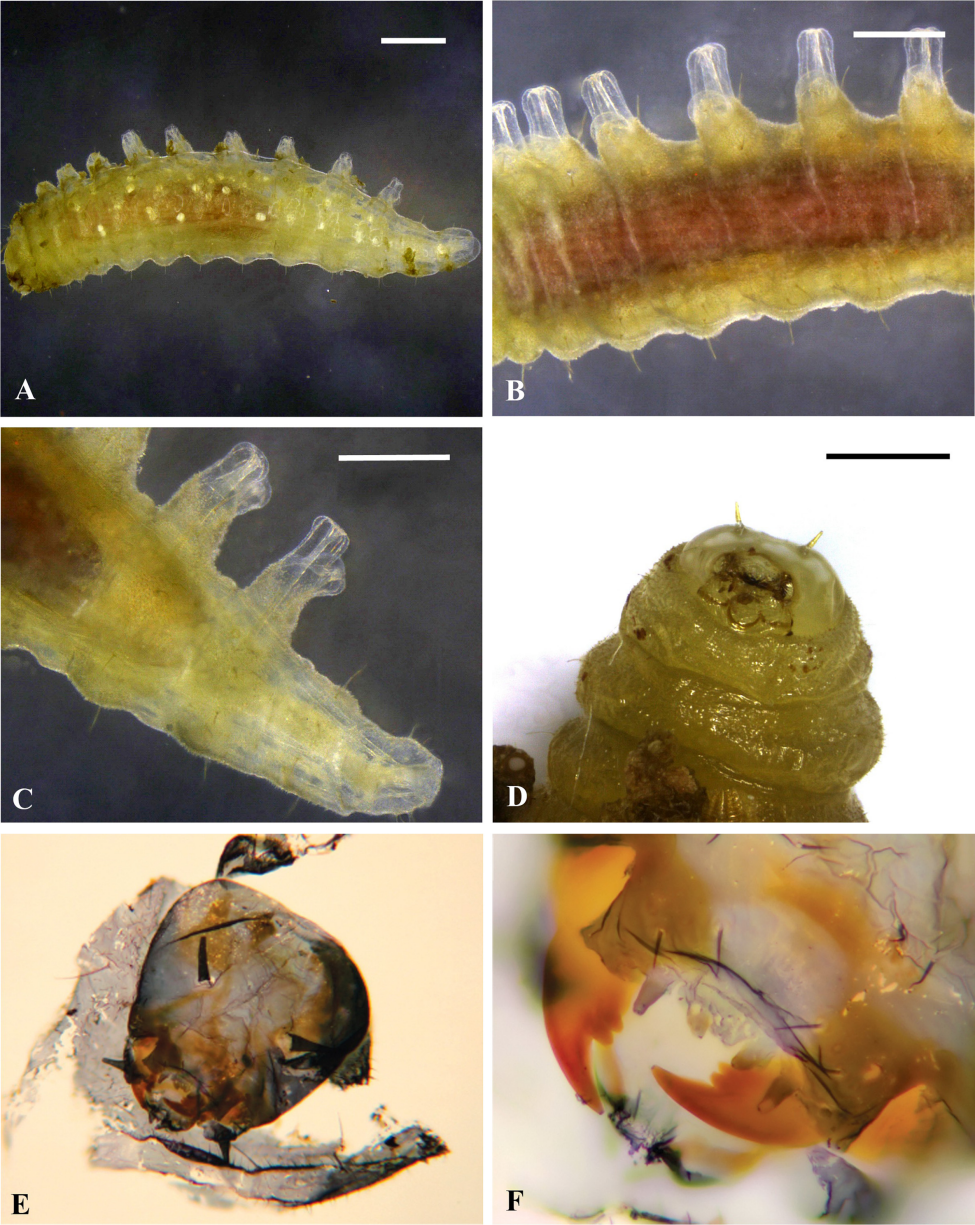
**Stereozoom and Light microscopic images of larva of *Bracon garugaphagae* Ranjith & Quicke sp. nov.** 2A–C, Mature larva showing dorsal abdominal tubercles with eversible tips.2D, Head capsule and anterior thorax of living mature larva showing denticulate cuticle. 2E, 2F, cl. Scale bars: (2A) 500 μm, (2B) 300 μm, (2C, 2D) 250 μm.

**Fig 3:**
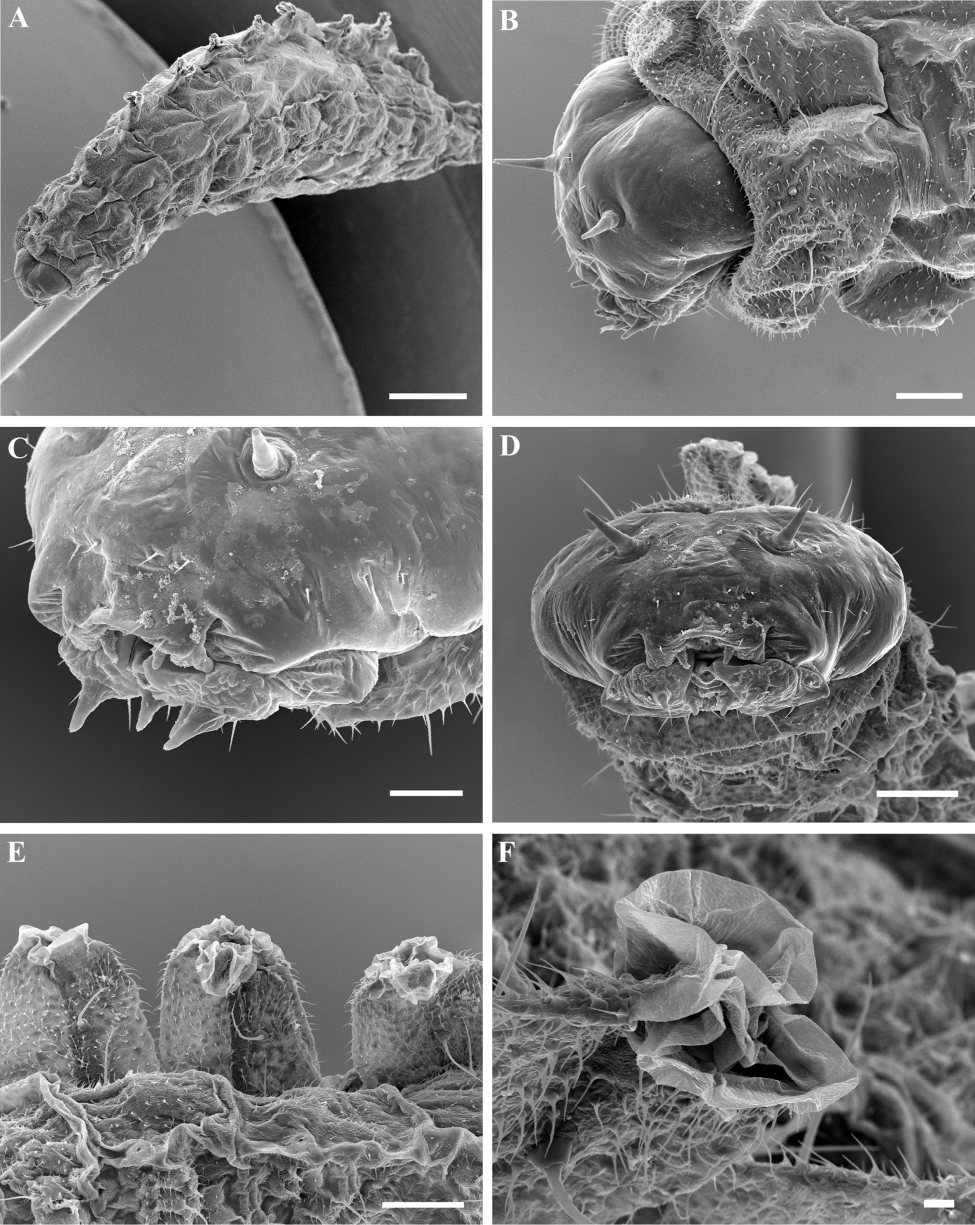
**Scanning electron microscope images of larva of *Bracon garugaphagae* Ranjith & Quicke sp. nov.** 3A–B, Final instar larva oblique dorsal view and detail of head capsule. 3C, Putative 2^nd^ instar larva detail of head capsule, 3D, Same, in anterior view, 3E, Dorsal, chimney-like tubercles on abdominal segments 1–9, 3F, Soft, eversible membranous lobes on the apex of tubercle. Scale bars: (A) 500 μm, (B, D, E) 100 μm, (C) 50 μm, (F) 10 μm.

**Fig 4:**
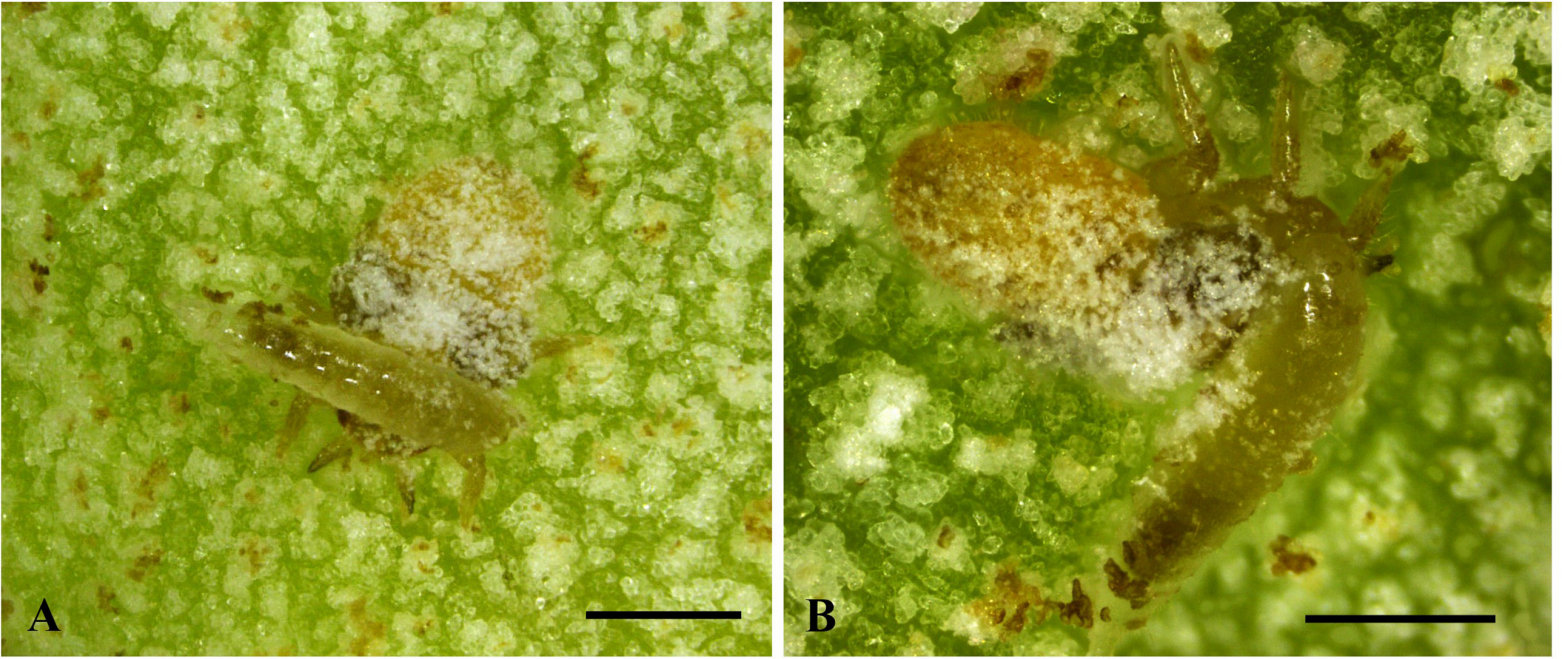
**Behaviour of *Bracon garugaphagae* Ranjith & Quicke sp. nov.** 4A–B, Braconid larva feeding on immature psyllids. Scale bars: (4A–B) 500 μm.

Larvae of all sizes possess unique, dorsal, chimney-like tubercles on abdominal segments 1–9 (Figs 2A, 2B, 2C, 3A–3E and 3F). The apex of each tubercle is formed of a pair of soft, eversible membranous lobes (Figs 2B, 2C and 3F) which help maintain the larva in position while feeding. The larval cuticle is extensively denticulate and the spiracular system open at all stages. The late instar larvae are covered with a white, waxy substance (Fig 4A and 4B). The larval head has well-developed papilliform antennae (Figs 2D, 2E, 3B, 3C and 3D) and labial and maxillary palps. The mandibles are strongly recessed, heavily sclerotised and possess two robust ancillary teeth near the base (Fig 2F). Most other cephalic structures are relatively weakly sclerotised (epistome, hypostome labial sclerite). Hypostomal spur virtually absent.

### Larval behaviour

Only one *Bracon* larva was observed inside each gall. *Bracon garugaphagae* preferentially attacks the third and fourth instar nymphs of the psyllid, by first coiling around the host and biting the host just below the head with their tridentate mandibles (Fig 4A and 4B), which results in the death of the host. Following feeding on the psyllid nymphs, *Bracon garugaphagae* larvae exhibit phytophagy which we observed directly in opened galls and confirmed by examining gut contents from mature larvae using both light and scanning electron microscopy. Plant tissue in the larval gut had been chewed into small irregular fragments; only rarely did we find any recognizable pieces of psyllid cuticle

### Incidence

The incidence of *B*. *garugaphagae* sp. nov. larvae in galls was monitored from August 2014 to January 2015. The larvae were first observed in galls which were 1–2 weeks old. The percentage incidence of braconid larvae in immature galls of size 8–12 mm in diameter was 6.5% (n = 779) and in mature galls of size 15–22 mm the incidence was 22%. Some of the galls (n = 53) were observed without any gall-inducing nymphs, but with the presence of *B*. *garugaphagae* larva.

**Fig 5:**
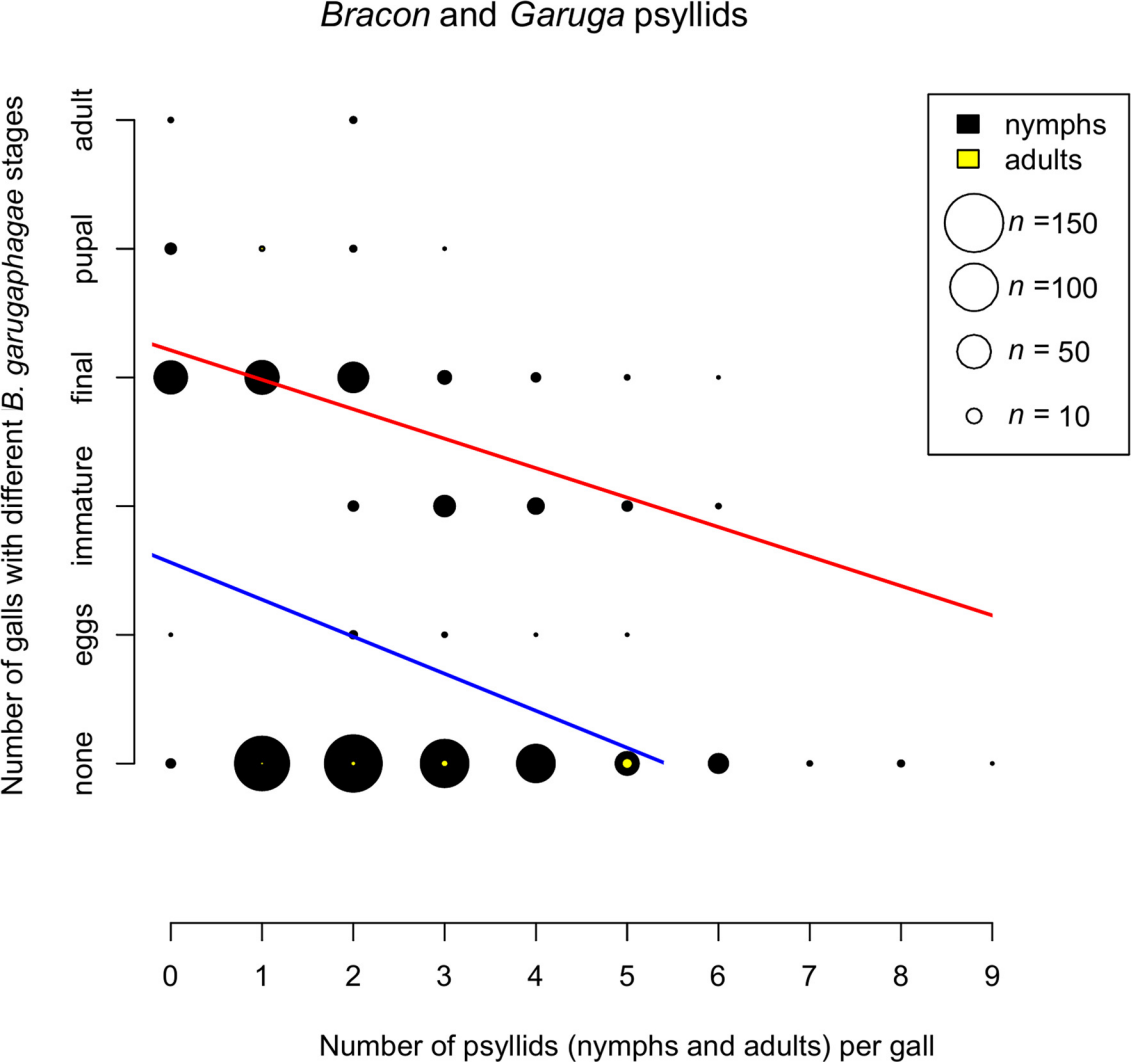
Relationship between the number of galls with different stages of *Bracon garugaphagae* Ranjith & Quicke sp. nov. and the number of live psyllids present in each gall. Symbol size indicates numbers of galls; numbers of galls with nymphal and adult psyllids are shown in black and yellow respectively. Regression lines are shown for all data (blue) and only data when *Bracon* present (red). Slopes of both relationships are highly significantly different from zero.

Fig 5 shows the relationship between the *Bracon* stages found in sampled galls and the number of psyllids present, both nymphs and adults. Two regression analyses were carried out, one with all galls included, even those that had no *Bracon* individuals present, and one with only galls containing a *Bracon* individual. Both relationships were highly significantly negative (all data: GLM with Poisson errors and log link-total data: null deviance = 674.62 on 778 degrees of freedom, residual deviance = 558.65 on 777 degrees of freedom, p<0.0001; only when *Bracon* present: null deviance = 30.195 on 248 degrees of freedom, residual deviance: 22.693 on 247 degrees of freedom, p<0.0001). Logistic regression of presence or absence of *Bracon* in galls (n = 779) versus number of psyllids present, showing highly significant negative relationship (GLM with binomial errors: null deviance = 976.24 on 778 degrees of freedom, residual deviance = 920.01 on 777 degrees of freedom, p<5e-12). Gall size and the presence or absence of *Bracon* were not correlated (GLM with binomial errors, p = 0.699), but there was a significant positive correlation between gall size and the number of psyllids (GLM with Poisson errors and log link: null deviance = 190.78 on 778 degrees of freedom, residual deviance = 188.82 on 777 degrees of freedom, p = 0.0046).

### Systematic part

**Fig 6:**
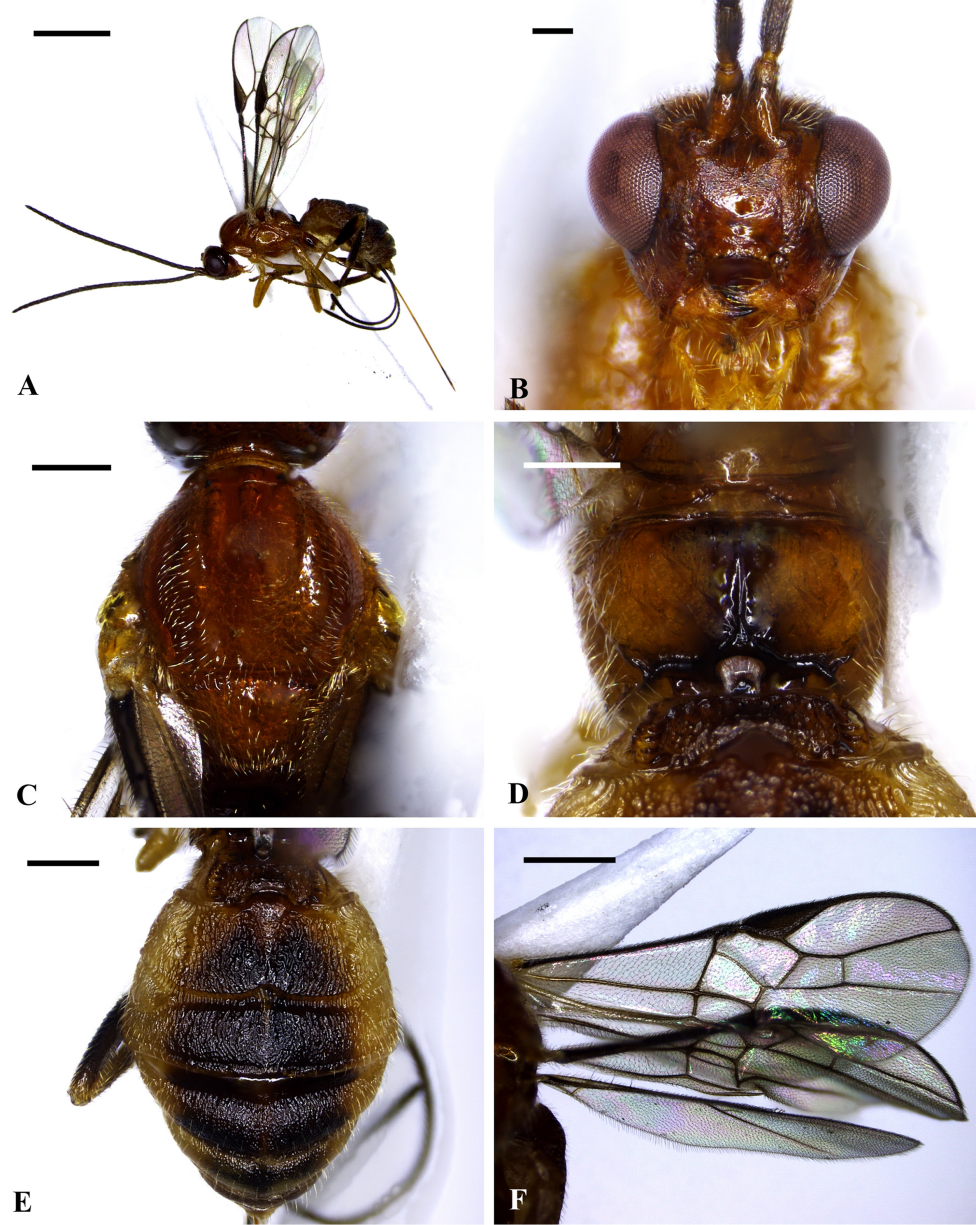
***Bracon garugaphagae* Ranjith & Quicke sp. nov., female, holotype;** 6A, Habitus in lateral view, 6B, Head in frontal view, 6C, Mesosoma in dorsal view, 6D, Propodeum and first metasomal tergite in dorsal view, 6E, Metasomal tergite in dorsal view, 6F, Wings. Scale bars: (6A) 1 mm, (B) 100 μm (6C, D, F) 200 μm (6E) 500 μm.

*Bracon garugaphagae* sp. nov. Ranjith & Quicke, 2015 (Fig 6)

urn:lsid:zoobank.org:act:AE7F1133-A2C2-4F16-A5BB-A660CF51C51F

#### Etymology

The new species is named after the plant on which it occurs.

#### Distribution

Known only from Kerala, south India.

Description: Female

Length of body 3.6 mm (3.6–5.8 mm in paratypes), of fore wing 2.8 mm (2.8–4 mm in paratypes), and of antenna 2.8 mm (2.8–3.8 mm in paratypes).

Head. Antenna with 24 flagellomeres (24–28 in paratypes). Terminal flagellomere strongly acute. Median flagellomeres normal in dorsal view. First flagellomere 1.2 times length of second and third flagellomeres respectively, first flagellomere 2.3 times as long as wide. Mandible twisted, only a single tooth visible in anterior view (Fig 6B). Inter-tentorial distance: tentorio-ocular distance = 1.5:1.0 (1.31–1.7: 0.82–1.07 in paratypes). Inter-tentorial distance: height of clypeus = 3.4: 1.0 (2.62–3.54: 0.81–1.04 in paratypes). Face slightly rugose in anterior half with smooth posterior half, sparsely setose laterally, smooth area laterally bordered by indistinct longitudinal groove (Fig 6B). Height of eye: shortest distance between eyes: width of head = 1.0: 1.1: 2.2(1.0–1.68: 1.1–1.83: 2.2–3.67 in paratypes). Oculo-antennal groove well-developed. Frons shiny. Stemmaticum triangular forming equilateral triangle. Shortest distance between posterior ocelli: transverse diameter of posterior ocellus: shortest distance between posterior ocellus and eye = 1.45: 1.0: 3.45 (1.45–1.81: 1–2.53: 3.45–7.06 in paratypes).

**Fig 7:**
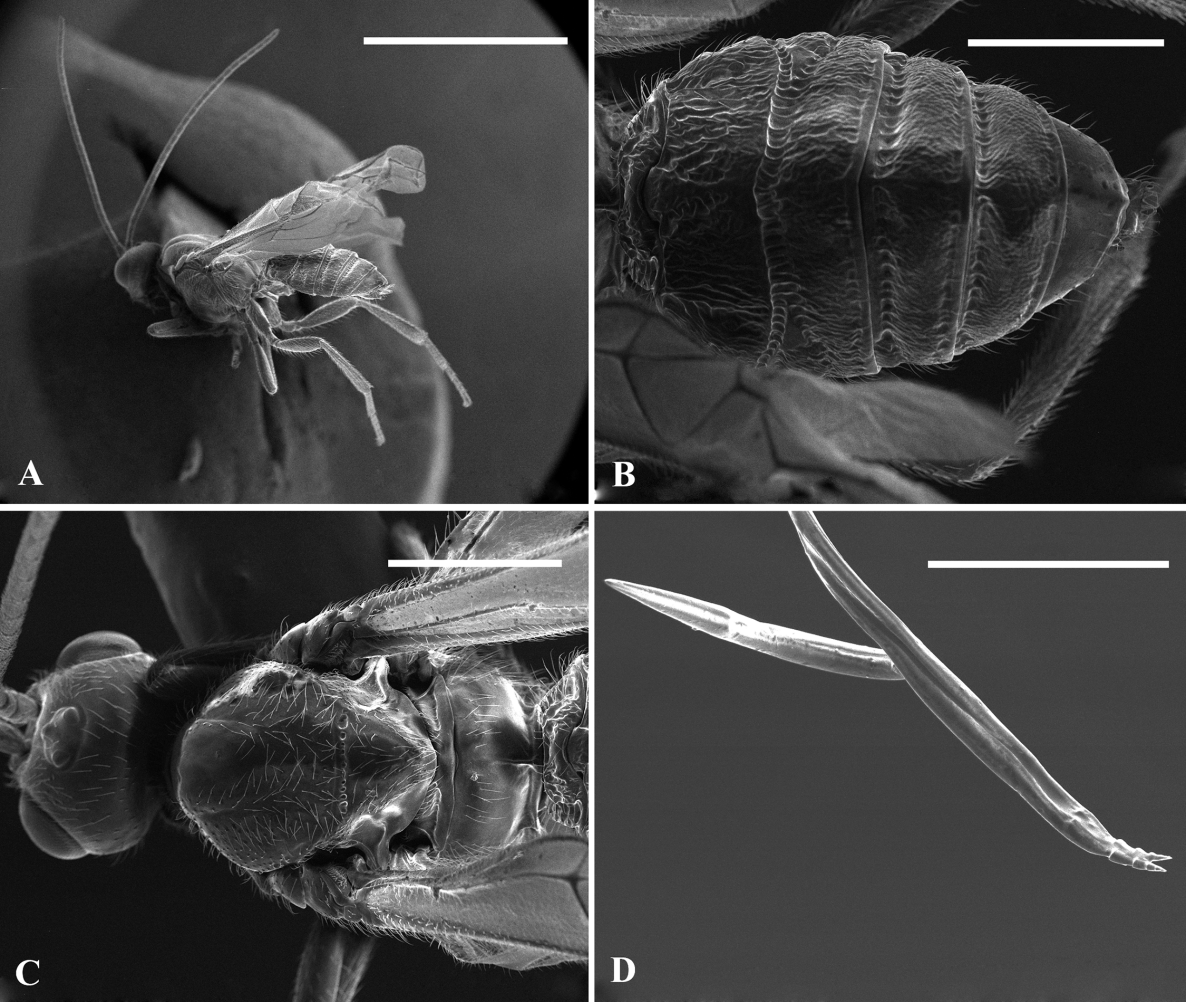
**SEM images of *Bracon garugaphagae* Ranjith & Quicke sp. nov., male, paratype;** 7A, Habitus in lateral view, 7B, Metasomal tergite in dorsal view, 7C, Head and mesosoma in dorsal view, 7D, Apex of ovipositor (female, paratype). Scale bars: (7A) 2 mm, (7B–C) 500 μm, (7D) 200 μm.

Mesosoma 1.6 (1.3–1.6 in paratypes) times longer than maximum height, largely smooth, shiny. Mesoscutum sparsely setose laterally (Fig 6C). Pronotum smooth. Notauli only indicated anteriorly (Figs 6C and 7C). Scutellar sulcus narrow, divided by eight carinae. Scutellum smooth (Figs 6C and 7C). Median area of metanotum large, smooth, slightly bulged in lateral view, without carina anteriorly. Propodeum smooth with a strong medial longitudinal carina (propodeal carina weak in paratypes), sparsely setose laterally (Fig 6D).

Fore wing, length of veins 3RSb: 3RSa: r-rs = 4.8: 1.7: 1.0 (4.8–5.1: 1.7–2: 1–1.3 in paratypes). Length of veins 2RS: 3RAa: rs-m = 1.6: 1.75: 1.0 (1.6–2.01: 1.75–2.14: 1–1.02 in paratypes). Vein 2-M 1.6 times 3RSa. Vein 1-M straight. Vein (RS+M)a strongly curved posteriorly (Fig 6F). Vein rs-m without bulla. Vein 1RS forming an angle of 70° with vein C+SC+R. Vein m-cu 0.46 times 1-M. Vein 1cu-a interstitial. Hind wing vein R1 = 1.4 (1.4–1.7 times in paratypes) times length of 1r-m. Apex of vein C+SC+R with one hamulus. Base of hind wing with medium sized glabrous area distal to vein cu-a on posterior half of cell.

Claws with pointed basal lobe. Lengths of fore femur: tibia: tarsus = 1.0: 1.1: 1.1 (1–1.3: 1.1–1.4: 1.1–1.3 in paratypes). Fore tibia with transverse apical row of thickened bristles. Lengths of hind femur: tibia: basitarsus = 1.8: 2.45: 1.0 (1.8–3.44: 2.45–4.52: 1–1.75 in paratypes).

Metasoma largely sculptured (moderately sculptured in paratypes) and dull with seven exposed, sparsely setose, tergites (Fig 6E). First metasomal tergite as long as wide, median area largely smooth and shiny, dorso-lateral carina strong and lamelliform. Second tergite rugose, 2.8 times wider than medially long, with large triangular mid basal area formed posteriorly into a mid-longitudinal carina that extends 0.3 times length of tergite, with a pair of sub lateral grooves. Second metasomal suture sinuate medially, strongly crenulate; third tergite rugose, 3.8 times wider than medially long, without sublateral grooves and with antero-lateral areas defined. Tergite 4–7 rugose, sparsely setose. Hypopygium sharply pointed, reaching end of metasomal tergites (Fig 6A). Ovipositor sheaths 2.2 times longer than hind tibia. Ovipositor slender, darkened sub apically (Fig 6A), dorsal valve with a distinct nodus and ventral valves with three normal teeth (Fig 7D).

Head brownish yellow except malar area, palps, basal region of mandible dark yellowish, tips of mandible black, antenna dark brown, compound eye greyish brown, mesosoma largely dark yellow except lateral mesoscutum brownish yellow, area near to medial longitudinal carina of propodeum dark brown, yellowish laterally, metasoma dark brown except lateral areas of tergites 1–6, medial area of first tergite yellow, mid basal area of second tergite dark yellow, tergites 2–6 blackish medially, hypopygium dark sub apically and ventrally, ovipositor sheath brown, legs mostly yellowish except fore telotarsi, hind coxa medially, hind femur, tibia and tarsus brown–black, wings hyaline, pterostigma dark brown, vein 1-SR+M and r-m less pigmented.

*Male*: Similar to female, somewhat smaller, 4–4.2 mm and antenna 2.8–3.1 mm, flagellum with 24 segments, body slightly yellower than females, and metasomal tergites less strongly sculptured (Fig 7A and 7B).

#### Material examined

*Holotype*: female, **INDIA, Kerala**, Malappuram, Kottakkal, 10°99’N, 76°00’E, emerged from leaf galls on *Garuga pinnata* Roxb. 20.ix.2014, leg. U.K.A. Saleem (Department of Zoology, University of Calicut, Kerala, India).

*Paratypes*: (12 females, 25 males), **INDIA, Kerala**, Malappuram, Kottakkal, 10°99’N, 76°00’E, emerged from leaf galls on *Garuga pinnata* Roxb. 1.x.2011, leg. U.K.A. Saleem (4 females, 20 males); same data except 20.ix.2014 (5 females, 4 males); **INDIA, Kerala**, Malappuram, Vettichira, 10°93’N, 76°02’E, emerged from leaf galls on *Garuga pinnata* Roxb. 21.i.2015, leg. A.P. Ranjith (3 females, 1 male).

All specimens were reared from leaf galls. The holotype and all paratypes are deposited in Department of Zoology, University of Calicut. Male and female pairs will be deposited in the Forest Research Institute, Dehradun, India (FRI), Natural History Museum, London, UK, the Smithsonian Institution, Washington DC, USA and Muséum National d'Histoire Naturelle, Paris, France.

#### Notes

This new species can be distinguished from the two other known phytophagous *Bracon* species, both of which are Neotropical, in having 24 flagellomeres (58 in *B*. *phytophagous* and 49 in *B*. *zuleideae*), median flagellomeres normal in dorsal view (diamond-shaped in *B*. *phytophagus*), face slightly rugose anteriorly and smooth posteriorly (smooth in *B*. *phytophagous* and *B*. *zuleideae*), scutellar sulcus moderately wide (narrow in *B*. *phytophagus*), propodeum with a strong medial longitudinal carina (propodeum smooth without carina in *B*. *phytophagous* and *B*. *zuleideae*), fore wing vein 1RS forming an angle of 70° with vein C+SC+R (80° in *B*. *phytophagous* and 100° *B*. *zuleideae*). Further, the Neotropical species are generally larger and have the ovipositor exceedingly thin and largely unsclerotised except for the apical part which is nearly black and presumably very hard.

*Bracon garugaphagae* sp. nov. can be distinguished from *B*. *psyllivorus* Achterberg (reared from psyllid-induced leaf galls) in having the frons with a distinct medial longitudinal suture, impressed notauli, scutellum without antero-medial puncture, scutellar sulcus divided by eight carinae (five in *B*. *psyllivorus*), metanotum not tuberculate in lateral view, metasomal tergites 3–5 with distinct longitudinal strip and apex of ovipositor with distinct dorsal nodus and ventral serrations.

Considering the size of the subfamily, very few braconines have sequence data available in GenBank. The closest BLAST search matches (conducted on 25/07/2015) for both sequenced gene fragments were members of the Braconinae but only 93% and 98% similar for CO1 and 28S respectively. *Bracon phytophagus* was not sequenced for CO1 but its 28S sequence is only 94% similar (differing in 29 positions) showing, as expected based on morphology, that the seed-predating New World species are only distantly related to the new Indian species. No DNA data are available for *B*. *psyllivorus* for comparison.

## Discussion

### Relationships of *Bracon garugaphagae* sp. nov

The new species belongs to the large spectrum of *Bracon* species of size between 3–6 mm and differs markedly from the two other known phytophagous *Bracon* species both of which are Neotropical [14, 15] notably in having the strongly sculptured metasomal tergites. DNA sequence data further indicates that they are only distantly related. Instead, the new species appears to be most closely related to *B*. *psyllivorus*, which also attacks gall-forming Psylloidea viz: *B*. *psyllivorus* reared from the leaf galls induced by *Pauropsylla gibberulosa* Li and *P*. *braconae* Li (Hemiptera: Triozidae) [18]. Apart from the host record, no further biological details are known for *B*. *psyllivorus* [18], and the possibility that it is also entomophytophagous cannot be excluded.

### Biology

Braconine wasps are almost entirely idiobiont ectoparasitoids of various concealed Coleoptera, Diptera, Hymenoptera and Lepidoptera with one small group of genera, the Aspidobraconina, being idiobiont endoparasitoids on exposed butterfly pupae [7, 31]. Psyllids have only been recorded as hosts of two other species of braconid wasp. Chadwick and Nikitin [32] recorded an unidentified *Bracon* sp. from a psyllid host in Australia, but no further details were provided. Recently, Li et al. [18] described a Chinese species, *B*. *psyllivorus*, as a parasitoid/predator of the psyllids *P*. *gibberulosa* Li and *P*. *braconae* Li (Hemiptera: Triozidae) that produce galls on the fig tree (*Ficus hainanensis* Merr. & Shun.; Moraceae).

The negative relationship between the number of psyllid individuals in galls and the developmental stage of *B*. *garugaphagae* indicates that the latter sequentially predate the psyllid nymphs before turning to phytophagy. The dietary shift seems likely to be because the psyllids do not provide *B*. *garugaphagae* with sufficient food to complete development. We do not know whether *B*. *garugaphagae* is obligately or only facultatively phytophagous during its late larval stages. In contrast to the previous explanation, the absence of *B*. *garugaphagae* larvae from galls with higher numbers (7–8 nymphs) of psyllids, may simply be that because the parasitoid larvae have to compete with psyllid nymphs for plant tissue the adult female parasitoids selectively avoid such galls.

Predatory behaviour is known in several parasitoid wasp taxa including cryptine ichneumonoids in spider egg sacs [7]. However, entomophytophagy whilst well-known in several eurytomid chalcidoids, which develop initially as parasitoids and complete their life cycle as phytophagous insects [33], was previously unknown in the Braconidae. Within the sister family Ichneumonidae there is evidence that at least one pimpline ichneumonid, a *Calliephialtes* species, does the same [Kenji Nishida, cited in 34]. Members of the labenine ichneumonid tribe Groteini consume the pollen ‘cake’ of their bee hosts after consuming the bee larva [35] and at least some *Poecilocryptus* species in the labenine tribe Poecilocryptini chew host gall tissue to reach other gall cells [36], but it is not confirmed whether any or all gain nutritive value from gall tissue though it seems likely. Within the Braconinae, there are a few other examples of predatory behaviour. One Neotropical species of *Compsobraconoides* is predatory on *Azteca* ants and their brood [37], an Afrotropical *Trigastrotheca* species also consumes ant broods in ant plant domatia [38]; and an Australian *Bracon* species consumes broods of gall-forming fergussoninid flies [32]. All these braconid species that prey on broods are contained within a single swollen plant structure, either an ant domatium or an insect-induced gall. It is also likely that a similar biology prevails in another braconine, *Ficobracon brusi* Achterberg & Weiblen, which has been reared from figs of *Ficus wassa* Roxb., but it is not clear whether it is a parasitoid/predator of the pollinating agaonid wasps (Chalcidoidea) or other fig inquilines or even whether it might be at least partly phytophagous [39]. Several other members of Braconinae, Doryctinae and Mesostoinae have also been associated with plant galls as inquilines or parasitoids on gall inducers [11, 39, 40] and the possibility that some of these are also partly phytophagous cannot be excluded.

Final instar larval cephalic structures, notably the long and relatively heavily sclerotised mandibles with two/three robust accessory teeth are very different from those of purely ectoparasitoid braconines. The latter have a very robust, almost square mandibular base and a short blade furnished with a series of small, closely spaced (comb-like) teeth [41]. The final instar larval mandibles of the entirely phytophagous *Bracon phylacteophagus* are even more heavily sclerotised, have even more closely-spaced robust ancillary teeth and the hypostomal spur is very well developed. In contrast, the mandibles of the gall-forming Mesostoinae are short-bladed, robust and lack ancillary teeth [42]. Clearly there are multiple possible types of larval head capsule adaptations for feeding on plant gall tissue.

All three known phytophagous *Bracon* species, viz., *B*. *phytophagus*, *B*. *zuleideae* and *B*. *garugaphagae* are associated with members of the plant family Burseraceae [14, 15]. The Neotropical species do not seem to induce gall tissue in the seeds they feed upon and their larval mandibles are far more highly modified and hardened than in the Indian species described here. The Neotropical species are not reliant on another insect having previously damaged the seed, and instead the apex of their ovipositors are extremely heavily sclerotised almost certainly as an adaptation to penetrating a much harder seed-coat. No such ovipositor modification is displayed by *B*. *garugaphagae* which only has to penetrate relatively softer gall tissue to reach its host.

## Supporting Information

S1 Video Showing the phytophagous feeding behaviour of *Bracon garugaphagae* Ranjith & Quicke sp. nov. (MP4). This video was recorded by A. P. Ranjith on June 2015 at the Insect Ecology and Ethology Laboratory, University of Calicut, with a Leica S8 APO stereozoomtrinocular microscope.
(MP4)
